# Mode of infant feeding, eating behaviour and anthropometry in infants at 6-months of age born to obese women – a secondary analysis of the UPBEAT trial

**DOI:** 10.1186/s12884-018-1995-7

**Published:** 2018-09-03

**Authors:** Nashita Patel, Kathryn V. Dalrymple, Annette L. Briley, Dharmintra Pasupathy, Paul T. Seed, Angela C. Flynn, Lucilla Poston, on behalf of the UPBEAT Consortium

**Affiliations:** Department of Women and Children’s Health, School of Life Course Sciences, Faculty of Life Sciences and Medicine, King’s College London, London, UK

**Keywords:** Infant growth patterns, Body composition, Breastfeeding, Appetite traits, Maternal obesity

## Abstract

**Background:**

Maternal obesity and rapid infant weight gain have been associated with increased risk of obesity in childhood. Breastfeeding is suggested to be protective against childhood obesity, but no previous study has addressed the potential benefit of breastfeeding as a preventive method of childhood obesity amongst obese women. The primary aim of this study was to assess the relationship between mode of feeding and body composition, growth and eating behaviours in 6-month-old infants of obese women who participated in UPBEAT; a multi-centre randomised controlled trial comparing a lifestyle intervention of diet and physical activity to standard care during pregnancy.

**Methods:**

Three hundred and fifty-three mother and infant pairs attended a 6-months postpartum follow-up visit, during which they completed the Baby-Eating Behaviour Questionnaire, a parent-reported psychometric measure of appetite traits. Measures of infant body composition were also undertaken. As there was no effect of the antenatal intervention on infant feeding and appetite the study was treated as a cohort. Using regression analyses, we examined relationships between: 1) mode of feeding and body composition and growth; 2) mode of feeding and eating behaviour and 3) eating behaviour and body composition.

**Results:**

Formula fed infants of obese women in comparison to those exclusively breastfed, demonstrated higher weight z-scores (mean difference 0.26; 95% confidence interval 0.01 to 0.52), higher rate of weight gain (0.04; 0.00 to 0.07) and greater catch-up growth (2.48; 1.31 to 4.71). There was also a lower enjoyment of food (*p* = 0.002) amongst formula fed infants, following adjustment for confounders. Independent of the mode of feeding, a measure of infant appetite was associated with sum of skinfold thicknesses (β 0.66; 95% CI 0.12 to 1.21), calculated body fat percentage (0.83; 0.15 to 1.52), weight z-scores (0.21; 0.06 to 0.36) and catch-up growth (odds ratio 1.98; 1.21 to 3.21).

**Conclusions:**

In obese women, exclusive breastfeeding was protective against increasing weight z-scores and trajectories of weight gain in their 6-month old infants. Measures of general appetite in early infancy were associated with measures of adiposity, weight and catch up growth independent of cord blood leptin concentrations and mode of early feeding.

**Electronic supplementary material:**

The online version of this article (10.1186/s12884-018-1995-7) contains supplementary material, which is available to authorized users.

## Background

Childhood obesity is a global pandemic; in 2016 more than 41 million children < 5 years of age were overweight or obese [1]. Strong observational evidence indicates that exposure to an adverse nutritional in-utero environment, arising from excessive maternal gestational weight gain (GWG) or maternal obesity, is associated with increased risk of obesity in the offspring [2–4]. In addition, the early infant growth trajectory has been linked to long term health [5, 6] as evidenced by the association between rapid early weight gain in the first few years of life and increased blood pressure [7], greater risk of obesity and the development of diabetes [8]. The trajectory of growth associated with the development of obesity in childhood may be established as early as 5 years of age in offspring born to mothers of heterogeneous body mass index (BMI) [9]. Recent observational studies have also demonstrated a role for early postnatal nutritional status and growth in the development of childhood obesity. These studies provide supporting evidence that early life mode and/or duration of feeding method may be a modifiable factor for optimising early growth trajectories [10, 11].

The World Health Organisation (WHO) recommend that all mothers exclusively breastfeed infants for the first 6 months of life and that breast feeding may protect against childhood obesity [12]. However, these guidelines are infrequently adhered to, especially in high income countries [13]. In the UK less than 1% of mothers are breastfeeding exclusively at 6-months postpartum [14] and obese women are less likely to initiate breastfeeding in comparison to their lean counterparts [15]. The low incidence and initiation of breastfeeding may therefore contribute to the development of obesity in children of obese mothers. Previously, research into early life feeding practices and infant growth has been undertaken in offspring born to women of heterogeneous BMI [16]. it is not known whether the maternal early feeding practices are also associated with infant body composition at 6-months of age in women with a high BMI.

Several studies have assessed eating styles and behaviours in infancy and their effect on weight development using the validated Baby Eating Behaviour Questionnaire (BEBQ) [17–19]. These have identified that increased appetite, specifically high food responsiveness and low satiety, are risk factors for rapid infant weight gain and subsequent childhood obesity. In relation to maternal obesity, evidence from experimental animals has suggested that a high maternal BMI is associated with reduced infant satiety, a relationship thought to be mediated by altered central pathways of energy regulation in the hypothalamus arising during fetal development [20]. If a similar relationship were to occur infants of obese women, in utero ‘programming’ of appetite could contribute to the reported relationship between maternal and offspring obesity.

We previously undertook a randomised controlled trial (RCT), the UK Pregnancies Better Eating and Physical Activity Trial (UPBEAT). UPBEAT was a multi-centre RCT comparing the effect of a lifestyle intervention of diet and physical activity advice compared to standard antenatal care in a group of obese pregnant women (*n* = 1555), from UK inner-city settings of ethnic and social diversity. The intervention focused on reducing dietary glycaemic load and saturated fat intake whilst increasing physical activity and was delivered from 15^+ 0^–18^+ 6^ weeks’ gestation for 8 weeks. The results of the study indicated that the intervention had no effect on the primary maternal or neonatal outcomes, incidence of gestational diabetes (GDM) and large for gestational age infants respectively, between the groups. However, there was a difference in secondary maternal outcomes including a reduction in GWG (− 0.55 kg; 95%CI -1.08 to − 0.02, *p* = 0.041), sum of skinfold thicknesses (− 3.2 mm, − 5.6 to − 0.8, *p* = 0.008) and an increase in physical activity at 28 weeks’ gestation (metabolic equivalent of task) (295; 95%CI 105 to 485, *p* = 0.0015) [21].

The primary aim of this study was to assess the role of mode of feeding, on measures of body composition, growth and eating behaviours in 6-month-old infants born to obese women. The investigation was a secondary analysis of the UPBEAT trial including 353 mother and infant dyads, all of whom had complete early feeding and body composition data as well as the parent-reported BEBQ. As no effect of the antenatal intervention was observed in any measures of infant feeding or appetite in this sub-group of participants, the data was treated as a cohort.

## Methods

Between July 2010 and May 2015, we conducted a planned follow-up at 6-months postpartum of infants and their mothers who had participated in the UPBEAT RCT. Women over the age of 16 years were recruited to the UPBEAT trial between 15^+ 0^ and 18^+ 6^ weeks’ gestation. The participants were from UK inner-city settings with high socioeconomic deprivation. Details of the trial intervention and inclusion and exclusion criteria have been published previously [21]. Mother and infant pairs were included within the present analysis (*n* = 353) if they had: 1) attended the follow-up visit at 6-months of age; 2) completed the feeding questionnaires; and 3) infant anthropometric data were obtained. Infants were excluded if they were suffering from major ill health which could impact on growth and development or born ≤34 weeks’ gestation.

### Infant variables

Data for mode, duration, type of milk and age of introduction of solids were collected using a validated feeding and growth questionnaire [22], administered by trained midwives at the 6-month postpartum visit. Modes of infant feeding were divided into three categories: 1) exclusive breastfeeding; breastfeeding for ≥4 months of age where infants received nothing else except water. (4 months is the most common time when women in the UK consider introduction of complementary foods [14]). 2) mixed feeding; formula feeding where the last episode of breastfeeding was between > 2 months and ≤ 4 months of age; and 3) formula feeding; infants receiving formula milk and, if applicable, where infants were exclusively breastfed before or until 2 months of age. Infants who received sugar sweetened beverages were classed as mixed feeding.

The validated 18-item Baby Eating Behaviour Questionnaire was used to assess infant appetite and feeding behaviours [18]. Mothers were required to score their baby’s feeding style during a ‘typical daytime feed’ and responses were on 5-point Likert scales for each item: never, rarely, sometimes, often, and always. The questionnaire has five distinctive appetite traits; enjoyment of food (EF), food responsiveness (FR), slowness in eating (SE), satiety responsiveness (SR) and general appetite [18]. Higher scores indicated greater appetite (higher EF, higher FR, faster eating, lower SR, and larger overall appetite).

### Maternal and neonatal variables

Maternal variables utilised in the present analysis included age, BMI, parity, ethnicity and socioeconomic status which were recorded at trial entry, and maternal GDM status, recorded following an oral glucose tolerance test at 24–28 week’s gestation. Neonatal data including gestational age at delivery and birthweight was collected at birth. In light of previously reported associations between cord blood leptin and infant adiposity [23], the leptin concentration obtained at delivery was also recorded and was treated as a confounder in the relevant analyses.

### Outcomes

The outcomes of interest were measures of infant adiposity assessed by subscapular and triceps skinfold thicknesses z-scores derived using the WHO reference populations [24]. Other outcomes included weight, length, BMI, mid-upper arm circumference z-scores, sum of skinfold thicknesses (mm), estimated body fat percentage derived from skinfold thicknesses and infant weight and length trajectory [24, 25]. Potential relationships between mode of feeding, catch-up growth, catch-down growth, weight and length trajectories from birth to 6-months of age were explored together with the incidence of overweight and obesity in infancy. For the purpose of these analyses, catch-up growth was defined as an increase of > 0.67 standard deviations of weight from birth to 6-months of age. Catch-down growth was defined as a decrease of > 0.67 standard deviations of weight from birth to 6-months of age [26].

### Statistical analysis

In this sub-group of participants, there was no effect of the antenatal intervention on any of the infant feeding or appetite variables (Additional file 1: Table S1), therefore the data was treated as a cohort. Treatment effects for continuous outcomes were expressed as differences in means obtained from multivariable linear regression, and binary endpoints as risk ratios with 95% confidence intervals obtained using binomial regression. This data is shown in Additional file 2: Table S2. Baseline maternal and infant characteristics were summarised by mode of early feeding: exclusive breastfeeding; formula feeding; and mixed feeding. Comparisons were made between these three categories and measures of infant body composition and anthropometry by chi-squared t-test, Anova or a Kruskal-Wallis-h-test, where appropriate. To investigate the associations between mode of feeding and measures of infant anthropometry and appetite and satiety, multiple linear or logistic regression were used, with adjustment for potential confounders and, where appropriate, exclusive breastfeeding was set as the reference category. Statistical significance for the interaction tests were defined as *p* < 0.05. Analyses were performed using Stata version 14.0 (StataCorp, College Station, TX, USA).

To estimate the effect of early mode of feeding on infant anthropometry, pre-defined adjustments were made for offspring sex, age at 6-month follow up visit and randomisation to the UPBEAT intervention (Model 1). Further adjustment was made for early pregnancy maternal BMI, ethnicity, index of multiple deprivation, incidence of GDM and birthweight (Model 2). To assess the influence of infant appetite and satiety on measures of adiposity and growth at 6-months of age, further adjustment was made for cord blood leptin concentration as leptin has been implicated in appetite regulation in offspring of obese mothers [27], and mode of infant feeding (Model 3).

**Fig. 1:**
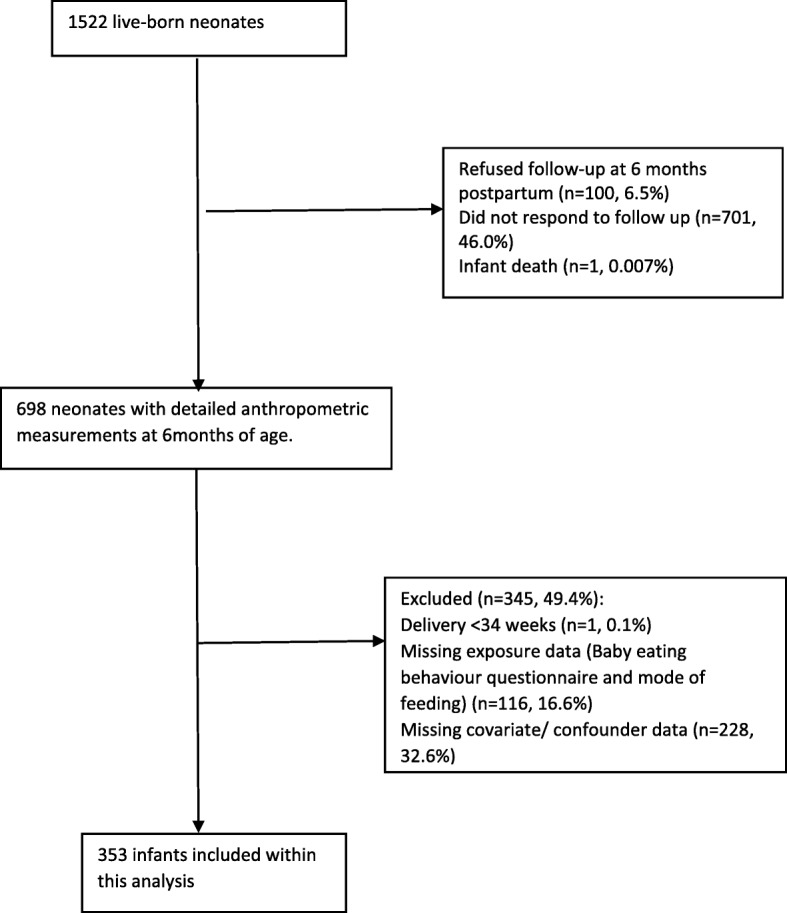
Flow diagram of mother-infant pairs included within this analysis

To assess for potential selection bias, comparisons were made between mother-offspring pairs included and excluded within this study. At the 6-month follow-up visit 698 infants had complete anthropometric data. Infants were excluded from the analysis due to missing mode of feeding and/or BEBQ data (*n* = 116) and confounder data (Additional file 3: Table S3) (*n* = 228) (Fig. 1). Three hundred and fifty-three complete data sets were included in the analysis. Assessment was made for the possibility that missing data for these exposures, or for any confounders, resulted in potential selection bias using three complementary methods. Firstly, the Little’s covariates dependent test was used to explore the potential of the data being missing not at random for missing exposure and confounder data in relation to infant outcomes at 6-months [28]. A second assessment was made, to identify predictors of missingness to determine whether the mechanism of missingness was missing completely at random or missing at random. Thirdly, as a sensitivity analysis to support the assessment of relationships between mode of feeding and infant growth and body composition in 353 infants with complete data, multivariate chain equations were used to impute the missing exposure and confounder data in all infants (*n* = 698). Assessment was undertaken to determine known predictors of missingness and data was subsequently imputed to create 50 datasets using 10-burn in iterations.

## Results

For the 698 infants who attended the 6-month visit, 353 with complete data were included in this analysis (Fig. 1). Of the included infants, 165 (47%) were exclusively breastfed ≥4 months, 161 (46%) were formula fed and 27 (7%) were fed a mixture of breastmilk, formula milk or sugar sweetened beverages (Table 1). In accord with previous data from women in the UK [29], the average weaning age was 4.6 months in this study, and introduction of solids was therefore subcategorised to “≤4.6 months” and “> 4.6 months”. Women who breastfed were more likely to be older and have a lower pre-pregnancy BMI compared to those of formula fed infants and were less likely to have had GDM in the index pregnancy (Table 1). There was no difference in distribution of infant anthropometry by offspring sex, and data was therefore not stratified by infant sex (Additional file 4: Figure S1).

**Table 1:** Maternal and neonatal demographic, clinical and biochemical characteristics stratified by mode of early feeding; data from the UPBEAT study (*n* = 353)

	Breast feeding *n* = 165	Formula feeding *n* = 161	Mixed feeding *n* = 27	*p*-value
	*Mean (SD)/ Median (IQR)/ N (%)*	*Mean (SD)/ Median (IQR)/N (%)*	*Mean (SD)/Median (IQR)/ N (%)*	
Maternal				
Age (years)	32.03 (4.72)	30.92 (5.45)	29.56 (5.55)	0.002
Multiparous	80 (48.48)	68 (40.48)	15 (53.57)	0.220
BMI (kg/m^2^)	35.87 (4.96)	37.30 (5.22)	35.06 (5.41)	0.01
Ethnicity				
White	101 (61.21)	131 (81.37)	19 (70.37)	< 0.001
Black	40 (24.24)	15 (9.32)	7 (25.93)	0.001
Asian	7 (4.24)	4 (2.48)	0 (0.00)	0.412
Other	17 (10.30)	11 (6.83)	1 (3.70)	0.352
Current smoker in early pregnancy	3 (1.82)	5 (3.11)	0 (0.00)	0.054
Socioeconomic deprivation	108 (78.36)	94 (77.05)	17 (85.00)	0.726
Gestational diabetes^a^	34 (20.61)	62 (38.51)	7 (25.93)	0.002
Gestational weight gain (kg)^b^	7.19 (4.39)	7.80 (4.75)	8.29 (4.37)	0.339
Neonate				
Gestation at delivery (weeks)	40.29 (39.00 to 41.00)	39.86 (38.43 to 41.71)	39.86 (38.86 to 41.00)	0.077
Birthweight (grams)	3600 (3210 to 3845)	3428 (3155 to 3760)	3360 (3140 to 3660)	0.159
Neonatal sum of skinfold thicknesses (mm)^	10.77 (2.81)	10.92 (2.91)	9.96 (1.78)	0.429
Neonatal abdominal circumference (cm)	32.17 (2.31)	32.30 (2.03)	32.02 (2.31)	0.867
Neonatal arm circumference (cm)	11.46 (0.99)	11.62 (1.00)	11.14 (0.83)	0.158
(log2) Cord blood leptin (ng/ml)	2.85 (0.70)	2.72 (0.72)	2.78 (0.71)	0.49

^a^Gestational diabetes diagnosed using the International Association of Diabetes in Pregnancy Group’s criteria at 24–28 weeks’ gestation. ^b^Gestational weight gain defined as total weight gain from calculated pre-pregnancy weight gain to 34–36 weeks’ gestation. ^Neonatal sum of skinfolds defined as sum of triceps skinfold thicknesses and subscapular skinfold thicknesses, each measured in triplicates. BMI, body mass index; breastfeeding defined as ≥4 months of age where infants received nothing else except water; formula feeding, defined as the last episode of exclusive breastfeeding ≤2 months of age. Data was also recorded for the age of introduction and type of formula milk provided. Mixed feeding was defined as the last episode of breastfeeding > 2 months and ≤ 4 months of age. In those breastfed infants who also received sugar sweetened beverages, this was classed as mixed feeding

The univariate analysis comparing mode of early life feeding and infant body composition are detailed in Additional file 1: Table S1. There was a significant difference for weight change kg/month for mode of feeding (*p* = 0.02). After adjustment for maternal and infant confounding factors detailed in Additional file 3: Table S3, formula feeding, in comparison to exclusive breastfeeding or mixed feeding, was associated with increased odds of catch-up growth (model 1 & 2, Table 2). Whilst model 1 (adjusted for intervention arm, infant sex and age) showed no association between mode of feeding and weight z-scores or rate of weight gain at 6-months of age, following further adjustment in model 2, formula feeding was associated with higher weight z-scores and rate of weight gain at 6-months of age, in comparison to infants exclusively breastfed or mixed fed (Table 2). Other measures of infant anthropometry did not differ by mode of early life feeding. Formula feeding in early life was associated with lower reported enjoyment of food (*p* = 0.002) in comparison to children who were breastfed (Table 3). Food responsiveness, general appetite, slowness in eating and satiety responsiveness did not differ between modes of feeding (Table 3).

**Table 2:** The role of mode of early feeding on measures of infant anthropometry at 6 months of age, in offspring born to obese women (*n* = 353)

		Breastfeeding	Formula feeding (*n* = 161)		Mixed feeding (*N* = 27)	
			*Mean difference (95% CI)*	*p-value*	*Mean difference (95% CI)*	*p-value*
Triceps SFT z-scores^a^	Model 1	REF	0.07 (−0.24 to 0.39)	0.64	0.00 (− 0.36 to 0.37)	0.98
	Model 2		0.20 (−0.39 to 0.79)	0.51	0.47 (− 0.19 to 1.13)	0.16
Subscapular SFT z-scores^a^	Model 1	REF	−0.10 (− 0.41 to 0.21)	0.53	0.21 (− 0.14 to 0.57)	0.23
	Model 2		0.03 (−0.55 to 0.62)	0.91	0.37 (−0.27 to 1.01)	0.25
Sum of skinfold thicknesses (mm)	Model 1	REF	−0.04 (− 0.92 to 0.84)	0.93	0.37 (−1.27 to 2.00)	0.66
	Model 2		−0.33 (− 0.64 to 1.29)	0.51	1.44 (− 0.30 to 3.19)	0.10
Total body fat estimation (%) ^	Model 1	REF	0.04 (−1.15 to 1.07)	0.94	0.47 (−1.61 to 2.54)	0.66
	Model 2		−0.44 (−0.78 to 1.65)	0.48	1.84 (−0.37 to 4.04)	0.10
Weight z-scores^a^	Model 1	REF	0.10 (−0.13 to 0.33)	0.39	0.05 (−0.38 to 0.47)	0.82
	Model 2		0.26 (0.01 to 0.52)	0.04**	0.21 (−0.25 to 0.67)	0.37
BMI z-scores^a^	Model 1	REF	0.13 (−0.24 to 0.51)	0.49	0.24 (−0.46 to 0.94)	0.50
	Model 2		0.23 (−0.21 to 0.67)	0.31	0.52 (− 0.28 to 1.32)	0.20
Length z-scores^a^	Model 1	REF	−0.01 (− 0.39 to 0.38)	0.97	− 0.34 (−1.05 to 0.38)	0.35
	Model 2		−0.26 (− 1.9 to 0.71)	0.26	− 0.30 (− 1.11 to 0.51)	0.46
Arm circumference z-scores ^a^	Model 1	REF	0.04 (− 0.18 to 0.27)	0.70	0.31 (−0.11 to 0.72)	0.15
	Model 2		0.10 (−0.16 to 0.36)	0.46	0.52 (0.05 to 1.00)	0.03**
Rate of weight gain (kg/ month)	Model 1	REF	0.03 (−0.00 to 0.05)	0.07	0.02 (−0.03 to 0.08)	0.39
	Model 2		0.04 (0.00 to 0.07)	0.04**	0.03 (−0.03 to 0.09)	0.41
Rate of length gain (cm/month)	Model 1	REF	0.07 (−0.10 to 0.25)	0.40	0.05 (−0.26 to 0.36)	0.76
	Model 2		0.15 (−0.07 to 0.37)	0.18	0.01 (−0.35 to 0.37)	0.96
BMI z-scores ≥85th ^a^	Model 1	REF	0.99 (0.48 to 2.05)	0.97	1.61 (0.49 to 5.27)	0.43
	Model 2		0.92 (0.35 to 2.40)	0.87	2.28 (0.54 to 9.65)	0.26
BMI z-scores ≥95th ^a^	Model 1	REF	1.58 (0.51 to 4.94)	0.43	2.40 (0.44 to 13.05)	0.31
	Model 2		2.45 (0.59 to 10.2)	0.22	2.22 (0.21 to 23.64)	0.51
Catch up growth ^a^	Model 1	REF	1.80 (1.10 to 2.92)	0.02**	1.71 (0.71 to 4.15)	0.24
	Model 2		2.48 (1.31 to 4.71)	0.01**	1.75 (0.59 to 5.25)	0.32
Catch down growth^a^	Model 1	REF	0.68 (0.41 to 1.13)	0.14	0.55 (0.19 to 1.54)	0.25
	Model 2		0.62 (0.32 to 1.21)	0.16	0.42 (0.10 to 1.75)	0.23

^a^Infant z-scores calculated using the WHO growth standards [24]. Catch up and catch down growth defined using the WHO definitions of change in weight > 0.67 SDs; Infant sum of skinfold thicknesses calculated as the addition of subscapular and triceps skinfolds thicknesses, each measured in triplicates. ^Infant total body fat estimation calculated sex-specific, validated equations [25]. Model 1- Adjustment made for randomisation to the UPBEAT Intervention, infant sex and infant age at anthropometric measurement. Model 2- Adjustment made randomisation to the UPBEAT Intervention, infant sex and infant age at anthropometric measurement as well as maternal early pregnancy BMI, ethnicity, socioeconomic deprivation, gestational diabetes and infant size at birth. ** *p* < 0.05. BMI, body mass index. Breastfeeding defined as ≥4 months of age where infants received nothing else except water; formula feeding, defined as the last episode of exclusive breastfeeding ≤2 months of age. Data was also recorded for the age of introduction and type of formula milk provided. Mixed feeding was defined as the last episode of breastfeeding > 2 months and ≤ 4 months of age. In those breastfed infants who also received sugar sweetened beverages, this was classed as mixed feeding

**Table 3:** Measures of infant appetite and satiety at 6 months of age by mode of early feeding in offspring born to obese women (*n* = 353)

	Breastfeeding	Formula feeding (N = 161)				Mixed feeding (N = 27)			
		Coef*	95% CI		*p*-value	Coef*	95% CI		*p*-value
			Lower limit	Upper limit			Lower limit	Upper limit	
Enjoyment of food	REF	−0.751	−1.235	−0.267	0.002	−0.909	−1.974	0.156	0.094
Food responsiveness	REF	0.365	−0.745	1.476	0.518	1.583	−4.222	3.588	0.121
General appetite	REF	−0.180	−0.441	0.081	0.176	0.043	−0.399	0.486	0.847
Slowness in eating	REF	−0.035	−0.578	0.507	0.898	0.380	−0.618	1.379	0.455
Satiety responsiveness	REF	0.371	−0.161	0.902	0.171	0.467	−0.607	1.541	0.393

Data obtained from the validated Baby Eating Behaviour Questionnaire [18]. *Adjustment made for randomisation to the UPBEAT intervention, infant sex, infant age at anthropometric measurement as well as maternal early pregnancy BMI, ethnicity, socioeconomic deprivation, gestational diabetes and infant size at birth. Breastfeeding defined as ≥4 months of age where infants received nothing else except water; formula feeding, defined as the last episode of exclusive breastfeeding ≤2 months of age. Data was also recorded for the age of introduction and type of formula milk provided. Mixed feeding was defined as the last episode of breastfeeding > 2 months and ≤ 4 months of age. In those breastfed infants who also received sugar sweetened beverages, this was classed as mixed feeding

**Fig. 2:**
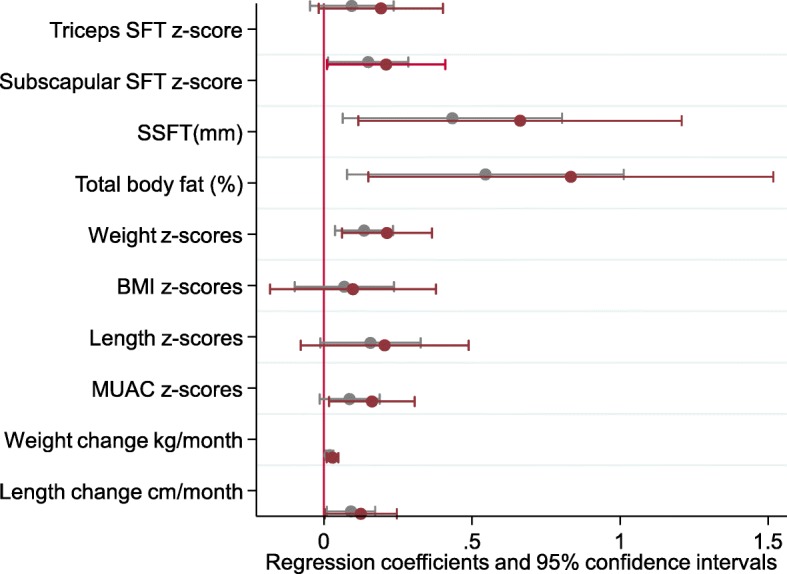
Associations between measures of general appetite with infant adiposity and anthropometry at 6 months of age, in offspring born to obese women (*n* = 353)

Independent of mode of feeding, a measure of appetite, assessed by the BEBQ, was positively associated with infant subscapular skinfold thickness (SFT), sum of skinfolds (SSFT), total body fat (%), weight z-scores and catch-up growth following adjustment for maternal and offspring confounders (Model 2) (Fig. 2, Additional file 5: Figure S2). Following adjustment for cord leptin concentrations and mode of feeding, the associations were strengthened (Model 3) (Fig. 2, Additional file 5: Figure S2). There were no associations between measures of enjoyment of food, food responsiveness or slowness of eating and infant growth or adiposity at 6-months of age (Additional file 6: Table S4).

Formula and mixed fed infants who were introduced to solids at ≤4.6 months of age had significantly higher length z-scores at 6-months than those breastfed and introduced solids > 4.6 months at 6-months (Additional file 7: Table S5). The relationship between mode of early feeding and other measures of infant body composition and infant anthropometry were not influenced by the timing of introduction of solids.

Assessment was made for selection bias by comparing baseline maternal and neonatal characteristics, it was found that mothers of infants included within the main analysis (*n* = 353) were significantly older, were more likely to be nulliparous and more likely to be white, in comparison to those excluded from the analysis (Additional file 8: Table S6). Neonates included in the study had a marginally lower, but significant, abdominal circumference than those excluded from the analysis at birth (Additional file 8: Table S6). Exclusion of infants born < 37 weeks’ gestation, was not associated with any significant changes in the observed associations (Additional file 9: Table S7).

To justify the decision for undertaking complete case analysis, we used Little’s Covariate dependent missing test, which demonstrated no evidence for the data being ‘missing not at random’ (Prob > Chi-square = 0.967). Centre, maternal age, number of years in full time education and gestation at delivery were identified to be significant predictors of missingness, suggesting missing data was ‘missing at random’ (Additional file 10: Table S8). Using multiple imputation by chained equations as a sensitivity analyses, there was no difference in the results obtained from complete-case analysis (*n* = 353) (Additional file 11: Table S9).

## Discussion

This study explored the associations of early life feeding practices on infant body composition, growth trajectories and eating behaviours in 6-month old infants of obese women. To our knowledge, this is the first study to analyse mode of feeding and appetite traits on infant growth patterns born to obese women and has provided some insight into associations between maternal obesity and childhood risk of adiposity amongst women drawn from inner city populations with high levels of socioeconomic deprivation. We have shown an association between exclusive breastfeeding and early-life infant growth patterns which has previously been implicated in the development of obesity in infants born to women with heterogeneous maternal BMI [30, 31]. Exclusive breastfeeding for more than 4 months was associated with infants having a higher enjoyment of food but with lower weight z-scores, trajectory of weight gain and catch-up growth. Conversely, this may suggest that the lower rates of breastfeeding previously reported in obese women [15] contribute to the reported risk of obesity in their children. We also report that in the infants of obese women, general appetite, regardless of mode of feeding, was associated with increased measures of adiposity, weight and catch-up growth, at 6-months of age. It follows, that amongst children of obese women, those with a greater appetite at 6 months may have a greater risk of lifelong obesity.

Whilst a number of studies have suggested that duration of breastfeeding is protective against obesity in childhood [31], we are not aware of any previous studies reporting outcomes in infants at 6-months of age confined to a cohort of obese women. Although there was no relationship between measures of adiposity and mode of feeding in these infants, exclusively breastfed infants had lower catch-up growth, weight z-scores and trajectory of weight gain in comparison to those who were formula fed. The lower catch-up and weight gain is likely to be attributable to different nutrient intakes; the low protein content of human milk compared to formula milk has been implicated in the protective effect of breastfeeding against later obesity in a recent review [32]. A higher energy intake in infants consuming formula milk may also contribute should these infants have a longer duration of feeding, as previously reported [33]. The demonstration that lower catch-up growth in infancy is protective against later obesity [5, 34], has contributed to a body of evidence suggesting that weight gain in the first few years of life is the best overall predictor of both later life obesity [34] and central fat distribution in both children and adolescents [5]. The lower growth trajectory reported here in obese women who breastfed could therefore be protective against childhood obesity. As reported in the UPBEAT cohort [35] and in others [36, 37] there is substantial evidence that obese women have either difficulty in initiating or maintaining breastfeeding. An additional benefit of encouraging breastfeeding amongst obese women, as suggested in the general population, could therefore be a reduction in the risk of childhood obesity.

Exclusively breastfed infants were more likely to demonstrate enjoyment of food as assessed at 6-months of age compared to formula or mixed fed infants. This observation has also been made recently in an Australian cohort of weight heterogeneous women (BMI 24 ± 5 kg/m^2^) using the same questionnaire [19]. This finding might seem unexpected as the term ‘enjoyment of food’ has previously been associated with the development of childhood obesity and therefore considered an ‘obesity risk’ characteristic [18]. However, breastfeeding in other studies has been associated with heightened slowness of eating [19, 38] which has been suggested to contribute to the lower risk of childhood obesity. However, in the present study there was no evidence for this association in breastfed infants of obese women compared with those mixed fed or formula fed. Whilst this requires repetition in other cohorts, this apparent difference in slowness of eating between breastfed infants of obese women and weight heterogeneous women could potentially blunt relationships between exclusive breastfeeding and infant adiposity observed in this study.

Surprisingly, one previous report has identified breastfeeding to be associated with reduced satiety responsiveness in weight heterogenous women [19], counterintuitive to the suggestion that breastfeeding is protective against excessive weight gain in the infant. In the present study, we did not observe any difference in satiety responsiveness between the different modes of feeding. There has however been some doubt about the validity of this element of the questionnaire [19].

The item ‘enjoyment of food’ in the BEBQ comprises the following questions: ‘my baby seems content while eating’, ‘my baby loves milk’, ‘my baby seems distressed while feeding’ (reverse scoring) and ‘my baby enjoys feeding time’. Rather than concluding that enjoyment of food is associated with greater food intake, an alternative explanation could be that the infants of obese women enjoyed human milk more than formula milk possibly due to a preferred odour of human milk [39]. Furthermore, the validation study for the BEBQ was completed in infants up to 8 months of age and the introduction of complementary foods may have altered the mother’s perception of their child’s eating behaviour in that study, limiting generalisability [18].

Early eating patterns and behaviours can determine later life eating habits and food preferences and have been associated with the development of childhood overweight and obesity [40, 41]. In the present study a measure of general appetite, assessed by the validated BEBQ, was found to be positively associated with measures of infant adiposity, weight z-scores and catch-up growth. This finding is similar to previous reports suggesting that the early postnatal environment, regardless of mode of feeding, may influence general appetite and the increased prevalence of obesity [42]. As far as we are aware this finding has previously not been reported in infants of obese women.

Strengths of the study include our sample of mothers and their infants are from a prospective cohort, recruited from UK inner-city populations. As 80% of the study population were in the highest quintile of socioeconomic deprivation and compromised entirely of obese women, this study is well placed to assess the mode of early feeding on infant anthropometric outcomes within this high-risk group amongst whom associations between maternal obesity and childhood obesity have frequently been described [43, 44]. Furthermore, due to the rich data set the observations made could be adjusted for a wide range of potential confounding variables increasing confidence in the observations made.

Limitations of the study include loss of follow-up of the study population which may result in selection bias and this limits transferability of findings to the general population. However, there were no differences in BMI, incidence of GDM or infant characteristics between those included and excluded within the analyses. Furthermore, complete outcome data was only available for 50% of the follow-up participants, however, sensitivity analyses including the use of multiple imputation did not result in differences in the observed relationships. The BEBQ is a parent-reported measure and subject to recall bias, it was used to assess appetite and satiety of the infant but did not distinguish between delivery of breastmilk via breast or bottle or volume of milk consumed; this may confound interpretation of results as previous studies have suggested that mode of delivery may influence appetite [45] and volume of milk consumed can vary between breast and formula fed infants [33]. The questionnaires also did not capture information regarding the mother’s decision to change from breastfeeding to formula feeding, which in obese women would be of particular interest in light of reported lower duration of breastfeeding [36]. Furthermore, the questionnaire was collected retrospectively, therefore if infants were older than 6-months at the study visit, the majority would have been weaned within this cohort. It could be suggested that it may be difficult for mothers to objectively assess two different modes of feeding retrospectively as well as potential subjective reporting and bias towards breastfeeding [18].

## Conclusion

In summary, exclusive breastfeeding in obese pregnant women modified early life childhood growth trajectories in infants at 6-months of age, in comparison to formula or mixed fed infants. Measures of general appetite in early infancy were also associated with measures of adiposity, weight and catch up growth in infants born to obese mothers from deprived inner-city UK populations. Given the association between maternal obesity and obesity in later life of the child these findings strongly support provision of lactation support for obese women, recognised to have difficulties in breastfeeding [15]. A novel and potentially important association between appetite and adiposity in 6-month infants of obese women not previously reported in any study of obese or weight heterogeneous women requires further investigation and replication with other birth cohorts. The results of the ongoing follow up of the UPBEAT children at 3 years are awaited with interest to provide further understanding of the long-term influence of early-life feeding practices on measures of body composition in early childhood.

## Additional files

Additional file 1: **Table S1.** Univariate analysis of infant body composition at 6 months of age stratified by mode of early feeding in offspring born to obese women (*n* = 353). (DOCX 14 kb)

Additional file 2: **Table S2**. Postnatal characteristics previously implicated with infant adiposity, by UPBEAT randomisation allocation. (DOCX 15 kb)

Additional file 3: **Table S3.** Description and reasoning behind potential confounders associated with mode of early feeding and infant anthropometry at 6 months of age [22, 46–53, 54–59]. (DOCX 14 kb)

Additional file 4: **Figure S1.** Distributional assessment of infant anthropometric measurements at 6 months of age by stratified by offspring sex. (DOCX 33 kb)

Additional file 5: **Figure S2.** Associations between measures of general appetite with infant obesity and growth at 6 months of age, in offspring born to obese women (*n* = 353). (DOCX 22 kb)

Additional file 6: **Table S4.** Associations between measures of infant appetite including measures of enjoyment of food, food responsiveness and slowness in eating with infant adiposity and anthropometry at 6 months of age, in offspring born to obese women (*n* = 353). (DOCX 15 kb)

Additional file 7: **Table S5.** The association between mode of early feeding, timing of introduction of solid foods and infant anthropometry at 6 months of age. (DOCX 15 kb)

Additional file 8: **Table S6.** Comparison of baseline maternal and neonatal demographic, clinical, and anthropometric characteristics between those included (*n* = 353) and excluded (*n* = 1167) from the analysis. (DOCX 15 kb)

Additional file 9: **Table S7.** Sensitivity analysis of removal of infants born > 34 weeks’ gestation and ≤ 37 weeks’ gestation (*n* = 8 excluded). (DOCX 14 kb)

Additional file 10: **Table S8**. Predictors of missing exposure (mode of infant feeding) and maternal covariate data in infants with detailed anthropometric data at 6 months of age. (DOCX 17 kb)

Additional file 11: **Table S9**. Sensitivity analyses assessing the role of mode of early feeding on measures of infant anthropometry at 6 months of age., in offspring born to obese women (*n* = 353) using multiple imputation. (DOCX 15 kb)

## Abbreviations

BEBQ: Baby eating behaviour questionnaire
BMI: Body mass index
GDM: Gestational diabetes mellitus
GWG: Gestational weight gain
RCT: Randomised controlled trial
SFT: Subscapular skinfold thickness
SSFT: Sum of skinfold thickness
UPBEAT: UK pregnancies better eating and physical activity trial
