# Supplementary material for: Mode of infant feeding, eating behaviour and anthropometry in infants at 6-months of age born to obese women – a secondary analysis of the UPBEAT trial
Source: BMC Pregnancy Childbirth. 2018 Sep 3;18:355. doi: 10.1186/s12884-018-1995-7 (PMC6122563; doi:10.1186/s12884-018-1995-7)
Supplement: Supplementary file 1 — Table S1. Univariate analysis of infant body composition at 6 months of age stratified by mode of early feeding in offspring born to obese women (n = 353). (DOCX 14 kb) [file 12884_2018_1995_MOESM1_ESM.docx]

| **Table S1: Univariate analysis of infant body composition at 6 months of age stratified by mode of early feeding in offspring born to obese women (n=353)** | | | | |
| --- | --- | --- | --- | --- |
|  | **Breastfeeding n=165** | **Formula feeding n=161** | **Mixed feeding n=27** | **p-value** |
|  | ***Mean (SD)/ N (%)*** | ***Mean (SD)/ N (%)*** | ***Mean (SD)/N (%)*** |  |
| Triceps skinfold z-scores* | 0.05 (1.50) | 0.16 (1.38) | 0.17 (1.30) | 0.79 |
| Subscapular skinfold z-scores* | 0.24 (1.45) | 0.16 (1.44) | 0.30 (1.42) | 0.82 |
| Sum of skinfold thickness (mm) | 17.30 (4.08) | 17.36 (3.94) | 17.50 (4.18) | 0.97 |
| Total body fat estimation (%) ^ | 19.75 (5.20) | 19.70 (5.06) | 19.81 (5.26) | 0.99 |
| Weight z-score* | 0.15 (1.06) | 0.28 (1.01) | 0.18 (1.04) | 0.52 |
| BMI z-scores* | -0.13 (1.85) | 0.06 (1.53) | 0.12 (1.44) | 0.68 |
| Length z-scores* | 0.51 (1.82) | 0.53 (1.60) | 0.18 (1.49) | 0.62 |
| Arm circumference z-scores * | 1.07 (1.01) | 1.14 (1.02) | 1.37 (1.06) | 0.39 |
| Weight change (kg/ month) | 0.63 (0.14) | 0.68 (0.13) | 0.67 (0.15) | 0.02 |
| Length change (cm/month) | 2.52 (0.64) | 2.64 (0.61) | 2.61 (0.54) | 0.38 |
| BMI z-scores ≥85^th^ * | 16 (9.7) | 15 (9.3) | 4 (14.8) | 0.61 |
| BMI z-scores ≥ 95^th^ * | 5 (3.0) | 8 (5.0) | 2 (7.4) | 0.47 |
| Catch up growth * | 38 (23.0) | 56 (34.8) | 9 (33.3) | 0.06 |
| Catch down growth* | 49 (29.7) | 36 (22.4) | 5 (18.5) | 0.23 |
| **Infant z-scores calculated using the WHO growth standards; Catch up and catch down growth defined using the WHO definitions of change in weight >0.67 SDs [24]. ^Infant total body fat estimation calculated sex-specific, validated equations [25].* | | | | |
