# Supplementary material for: Mode of infant feeding, eating behaviour and anthropometry in infants at 6-months of age born to obese women – a secondary analysis of the UPBEAT trial
Source: BMC Pregnancy Childbirth. 2018 Sep 3;18:355. doi: 10.1186/s12884-018-1995-7 (PMC6122563; doi:10.1186/s12884-018-1995-7)
Supplement: Supplementary file 2 — Table S2. Postnatal characteristics previously implicated with infant adiposity, by UPBEAT randomisation allocation. (DOCX 15 kb) [file 12884_2018_1995_MOESM2_ESM.docx]

| **Table S2:** **Postnatal characteristics previously implicated with infant adiposity, by UPBEAT randomisation allocation.** | | | | | | |
| --- | --- | --- | --- | --- | --- | --- |
|  | **Intervention** | | **Control** | | **Diff/ Risk ratio*** | **p-value** |
|  | **Mean (SD)/N(%)** | | **Mean (SD)/N(%)** | |  |  |
| **Infant feeding at 6 months** | | | | | | |
| Breast milk only | N=336 | 9 (2.7) | N=347 | 10 (2.9) | 1.01 (0.42 to 2.43) | 0.982 |
| Mixed feeding | N=336 | 55 (16.4) | N=347 | 62 (17.9) | 0.97 (0.70 to 1.33) | 0.830 |
| Breast milk &solids | N=336 | 57 (17.0) | N=347 | 61 (17.6) | 0.98 (0.71 to 1.36) | 0.917 |
| Formula & solids | N=336 | 193 (57.4) | N=347 | 193 (55.6) | 1.05 (0.93 to 1.19) | 0.400 |
| Formula only | N=336 | 22 (6.5) | N=347 | 21 (6.1) | 1.09 (0.62 to 1.95) | 0.759 |
| Days exclusively breast fed | N=260 | 80.57 (65.11) | N=243 | 85.04 (65.60) | -4.81 (-16.18 to 6.57) | 0.407 |
| **Appetite and satiety **** | | | | | | |
| Enjoyment of food | N=293 | 18.54 (2.23) | N=314 | 18.40 (2.45) | 0.15 (-0.22 to 0.52) | 0.425 |
| Food responsiveness | N=342 | 11.94 (4.95) | N=350 | 12.34 (4.87) | -0.39 (-1.12 to 0.33) | 0.288 |
| General appetite | N=342 | 3.56 (1.31) | N=350 | 3.65 (1.18) | -0.08 (-0.27 to 0.10) | 0.382 |
| Slowness in eating | N=342 | 10.08 (2.62) | N=350 | 10.26 (2.55) | -0.18 (-0.56 to 0.20) | 0.353 |
| Satiety responsiveness | N=341 | 6.49 (2.63) | N=350 | 6.46 (2.48) | 0.02 (-0.36 to 0.40) | 0.907 |

**Treatment effect adjusted for minimisation variables of randomisation (maternal BMI, ethnicity and parity), infant age at 6 month follow up and infant sex.** Appetite and satiety assessed by the Baby Eating Behaviour Questionnaire [18]*
