# Supplementary material for: Mode of infant feeding, eating behaviour and anthropometry in infants at 6-months of age born to obese women – a secondary analysis of the UPBEAT trial
Source: BMC Pregnancy Childbirth. 2018 Sep 3;18:355. doi: 10.1186/s12884-018-1995-7 (PMC6122563; doi:10.1186/s12884-018-1995-7)
Supplement: Supplementary file 3 — Table S3. Description and reasoning behind potential confounders associated with mode of early feeding and infant anthropometry at 6 months of age [22, 46–53, 54–59]. (DOCX 14 kb) [file 12884_2018_1995_MOESM3_ESM.docx]

| **Table S3: Description and reasoning behind potential confounders associated with mode of early feeding and infant anthropometry at 6 months of age.** | | |
| --- | --- | --- |
| **Confounder** | **Definition** | **Reasoning** |
| Maternal age | Continuous | Increasing maternal age has been associated with increasing duration of exclusive breastfeeding [46]. Increasing maternal age, is associated with a taller stature and reduced abdominal adiposity in infant aged between 19-44 years [47]. |
| Maternal BMI | Continuous | Increasing maternal BMI is an independent risk factor for early introduction of solid food in comparison to infants of lean women [48]. Maternal BMI has been identified as an independent risk factor for increasing infant adiposity [44, 49]. |
| Parity | Binary; 0-Nultip (reference), 1-Multip | Multiparty is associated with increased rates of being exclusive breastfeeding duration [50] as well as independently associated with increasing infant adiposity [51]. |
| Maternal socioeconomic status | Binary; 0-No socioeconomic deprivation (reference), 1-Socioeconomic deprivation | Population level data has identified associations between sociodemographic level and breastfeeding [52]. Increasing sociodemographic deprivation has been previously associated with increased risk of childhood obesity [53]. |
| Maternal ethnicity | Categorical; 0-White (reference), 1-Black, 2-Asian, 3-Other | Maternal ethnicity is strongly associated with infant adiposity. Furthermore, maternal ethnicity influences the immediate familial environment subsequently influencing feeding practices within the household [54]. |
| Maternal educational attainment | Binary; 0 <12 years full time education, 1->12 years of full time education (reference) | Reduced educational attainment has been associated with reduced duration of exclusive breastfeeding. Educational attainment is an indicator of parental social class. Observational studies have identified a dose response relationship between educational attainment and childhood obesity [55]. |
| Size at birth | Birthweight/ birthweight z-scores | Size at birth is a known predictor of later obesity throughout the life course [56]. Infants with higher birthweights have previously been shown to have larger appetites, fed more, had greater enjoyment of food and increased responsiveness to food cues in comparison to low birthweight infants [18, 57]. |
| Maternal gestational diabetes | Binary; 0- No gestational diabetes (reference), 1- Gestational diabetes | Maternal gestational diabetes has been previously associated with infant body composition [58]. Furthermore, gestational diabetes has been shown to influence the mode of early feeding including reduced rates of initiation of breastfeeding [59]. |
| Total gestational weight gain | Continuous measured in Kg | Total gestational weight gain has been identified as a significant predictor of infant body composition independently as well as mediated through cord leptin [60]. |
