# Supplementary material for: Mode of infant feeding, eating behaviour and anthropometry in infants at 6-months of age born to obese women – a secondary analysis of the UPBEAT trial
Source: BMC Pregnancy Childbirth. 2018 Sep 3;18:355. doi: 10.1186/s12884-018-1995-7 (PMC6122563; doi:10.1186/s12884-018-1995-7)
Supplement: Supplementary file 4 — Figure S1. Distributional assessment of infant anthropometric measurements at 6 months of age by stratified by offspring sex. (DOCX 33 kb) [file 12884_2018_1995_MOESM4_ESM.docx]

**Figure S1: Distributional assessment of infant anthropometric measurements at 6 months of age by stratified by offspring sex.**

*Abbreviations: AbdominalC- Abdominal circumference, ArmC-Arm circumference*
