# Supplementary material for: Mode of infant feeding, eating behaviour and anthropometry in infants at 6-months of age born to obese women – a secondary analysis of the UPBEAT trial
Source: BMC Pregnancy Childbirth. 2018 Sep 3;18:355. doi: 10.1186/s12884-018-1995-7 (PMC6122563; doi:10.1186/s12884-018-1995-7)
Supplement: Supplementary file 5 — Figure S2. Associations between measures of general appetite with infant obesity and growth at 6 months of age, in offspring born to obese women (n = 353). (DOCX 22 kb) [file 12884_2018_1995_MOESM5_ESM.docx]

*
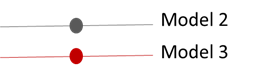
*

***Figure S2: Associations between measures of general appetite with infant obesity and growth at 6 months of age, in offspring born to obese women (n=353).***

*Odds ratio and 95% confidence intervals plotted. Model 2: adjustment made for randomisation to the UPBEAT intervention, maternal early pregnancy BMI, and socioeconomic deprivation, and ethnicity, diagnosis of gestational diabetes, offspring birthweight, sex and age at 6 month follow-up visit. Model 3- adjustment made for randomisation to the UPBEAT intervention, maternal early pregnancy BMI, socioeconomic deprivation, ethnicity, diagnosis of gestational diabetes, offspring birthweight, sex, cord blood leptin, age at 6 month follow-up and mode of early feeding. Abbreviations BMI-Body mass index, catch up and catch down growth defined using the WHO definitions of change in weight >0.67 SDs*
