# Supplementary material for: Mode of infant feeding, eating behaviour and anthropometry in infants at 6-months of age born to obese women – a secondary analysis of the UPBEAT trial
Source: BMC Pregnancy Childbirth. 2018 Sep 3;18:355. doi: 10.1186/s12884-018-1995-7 (PMC6122563; doi:10.1186/s12884-018-1995-7)
Supplement: Supplementary file 6 — Table S4. Associations between measures of infant appetite including measures of enjoyment of food, food responsiveness and slowness in eating with infant adiposity and anthropometry at 6 months of age, in offspring born to obese women (n = 353). (DOCX 15 kb) [file 12884_2018_1995_MOESM6_ESM.docx]

| **Table S4: Associations between measures of infant appetite including measures of enjoyment of food, food responsiveness and slowness in eating with infant adiposity and anthropometry at 6 months of age, in offspring born to obese women (n=353).** | | | |
| --- | --- | --- | --- |
|  | **Enjoyment of food** | **Food responsiveness** | **Slowness in eating** |
|  | ***Mean difference (95% CI)/***  ***Odds ratio (95% CI) †*** | ***Mean difference (95% CI)/***  ***Odds ratio (95% CI) †*** | ***Mean difference (95% CI)/***  ***Odds ratio (95% CI) †*** |
| Triceps skinfold z-scores * | -0.04 (-0.19 to 0.10) | 0.02 (-0.03 to 0.07) | -0.04 (-0.15 to 0.06) |
| Subscapular skinfold thickness z-scores* | -0.05 (-0.19 to 0.09) | 0.03 (-0.01 to 0.08) | -0.01 (-0.11 to 0.09) |
| Sum of skinfold thickness (mm)** | -0.10 (-0.48 to -0.28) | 0.08 (-0.47 to 0.21) | -0.10 (-0.37 to 0.17) |
| Total body fat estimation (%) ^ | -0.12 (-0.60 to 0.36) | 0.10 (-0.05 to 0.26) | -0.13 (-0.47 to 0.21) |
| Weight z-scores* | -0.07 (-0.18 to 0.03) | 0.02 (-0.02 to 0.06) | -0.02 (-0.09 to 0.06) |
| BMI z-scores * | -0.06 (-0.26 to 0.13) | -0.08 (-0.07 to 0.06) | -0.12 (-0.26 to 0.02) |
| Length z-scores* | -0.05 (-0.25 to 0.15) | 0.03 (-0.03 to 0.10) | 0.08 (-0.05 to 0.22) |
| Arm circumference z-scores * | -0.06 (-0.16 to 0.04) | 0.01 (-0.03 to 0.04) | -0.03 (-0.10 to 0.04) |
| Weight change (kg/month) | -0.01 (-0.02 to 0.00) | 0.00 (-0.00 to 0.01) | -0.00 (-0.01 to 0.01) |
| Length change (cm/month) | -0.04 (-0.12 to 0.05) | 0.15 (-0.01 to 0.43) | 0.04 (-0.01 to 0.10) |
| BMI z-scores ≥85^th^ centile | 0.86 (0.63 to 1.18) | 1.03 (0.88 to 1.20) | 0.72 (0.51 to 1.04) |
| BMI z-scores ≥95^th^ centile | 1.02 (0.51 to 2.06) | 0.78 (0.57 to 1.10) | 0.57 (0.29 to 1.11) |
| Catch up growth** | 0.98 (0.76 to 1.26) | 1.13 (0.98 to 1.26) | 0.88 (0.71 to 1.07) |
| Catch down growth** | 1.10 (0.78 to 1.55) | 0.98 (0.90 to 1.08) | 1.01 (0.81 to 1.25) |
| *No significant associations were observed between measures of enjoyment of food, food responsiveness and slowness of eating with infant anthropometry at 6 months of age. Data obtained from the validated Baby Eating Behaviour Questionnaire [18] *Infant z-scores calculated using the WHO growth standards [24]; **Catch up and catch down growth defined using the WHO definitions of change in weight >0.67 SDs; Infant sum of skinfold thicknesses calculated as the addition of subscapular and triceps skinfolds thicknesses, each measured in triplicates. ^Infant total body fat estimation calculated sex-specific, validated equations [25]. † Analyses adjusted for randomisation to the UPBEAT intervention, maternal early pregnancy BMI, socioeconomic deprivation, ethnicity, and diagnosis of gestational diabetes, offspring birthweight, sex, and cord blood leptin, age at 6 month follow-up and mode of early feeding, cord leptin and mode of early feeding.* | | | |
