# Supplementary material for: Mode of infant feeding, eating behaviour and anthropometry in infants at 6-months of age born to obese women – a secondary analysis of the UPBEAT trial
Source: BMC Pregnancy Childbirth. 2018 Sep 3;18:355. doi: 10.1186/s12884-018-1995-7 (PMC6122563; doi:10.1186/s12884-018-1995-7)
Supplement: Supplementary file 7 — Table S5. The association between mode of early feeding, timing of introduction of solid foods and infant anthropometry at 6 months of age. (DOCX 15 kb) [file 12884_2018_1995_MOESM7_ESM.docx]

| Table S5: The association between mode of early feeding, timing of introduction of solid foods and infant anthropometry at 6 months of age. | | | | |
| --- | --- | --- | --- | --- |
|  | **Breastfeeding** | **Formula feeding solids<4.6months (n=45)** | **Mixed feeding solids<4.6months (N=10)** | **p-value** |
|  |  | Mean difference (95% CI) † | Mean difference (95% CI) † |  |
| Triceps skinfold z-scores * | REF | 0.47 (-1.12 to 2.06) | -1.41 (-4.34 to 1.52) | 0.43 |
| Subscapular skinfold thickness z-scores* | REF | -0.12 (-1.67 to 1.43) | -1.71 (-4.58 to 1.15) | 0.48 |
| Sum of skinfold thickness (mm)* | REF | 0.35 (-3.51 to 4.21) | -5.45 (-12/54 to 1.70) | 0.27 |
| Total body fat estimation (%) ^ | REF | 0.36 (-4.45 to 5.17) | -6.74 (-15.62 to 2.14) | 0.27 |
| Weight z-scores* | REF | 0.71 (-0.40 to 1.83) | 0.27 (-1.79 to 2.34) | 0.44 |
| BMI z-scores * | REF | 0.37 (-1.15 to 1.89) | -2.37 (-5.17 to 0.43) | 0.16 |
| Length z-scores* | REF | 0.79 (-0.58 to 2.16) | 3.25 (0.75 to 5.75) | 0.04** |
| Arm circumference z-scores * | REF | 0.67 (-0.46 to 1.80) | -0.75 (-2.84 to 1.33) | 0.28 |
| Weight change (kg/month) | REF | 0.10 (-0.49 to 0.25) | 0.04 (-0.23 to 0.32) | 0.41 |
| Length change (cm/month) | REF | -0.13 (-0.63 to -0.36) | 0.80 (-0.10 to 1.71) | 0.11 |
| BMI z-scores ≥85^th^ * | REF | - | - | - |
| BMI z-scores ≥ 95^th^ * | REF | - | - | - |
| Catch up growth | REF | 0.88 (0.11 to 6.87) | 1.01 (0.10 to 9.86) | 0.871 |
| Catch down growth | REF | 0.70 (0.079 to 6.14) | 1.77 (0.10 to 32.70) | 0.611 |
| Breastfeeding and introduction of solids ≥4.6 months was treated the reference category within the analysis. **Infant z-scores calculated using the WHO growth standards [24];* *Catch up and catch down growth defined using the WHO definitions of change in weight >0.67 SDs;* Infant sum of skinfold thicknesses calculated as the addition of subscapular and triceps skinfolds thicknesses, each measured in triplicates. ^Infant total body fat estimation calculated sex-specific, validated equations [25]. †Analyses adjusted for randomisation to the UPBEAT Intervention, infant sex and infant age at anthropometric measurement as well as maternal early pregnancy BMI, ethnicity, socioeconomic deprivation, gestational diabetes and infant size at birth. **p<0.05. | | | | |
