# Supplementary material for: Mode of infant feeding, eating behaviour and anthropometry in infants at 6-months of age born to obese women – a secondary analysis of the UPBEAT trial
Source: BMC Pregnancy Childbirth. 2018 Sep 3;18:355. doi: 10.1186/s12884-018-1995-7 (PMC6122563; doi:10.1186/s12884-018-1995-7)
Supplement: Supplementary file 8 — Table S6. Comparison of baseline maternal and neonatal demographic, clinical, and anthropometric characteristics between those included (n = 353) and excluded (n = 1167) from the analysis. (DOCX 15 kb) [file 12884_2018_1995_MOESM8_ESM.docx]

| **Table S6: Comparison of baseline maternal and neonatal demographic, clinical, and anthropometric characteristics between those included (n=353) and excluded (n=1167) from the analysis.** | | | |
| --- | --- | --- | --- |
|  | **Included** | **Excluded** | **P-value** |
|  | ***Mean (SD)/ Median (IQR)/ N (%)***  ***N=353*** | ***Mean (SD)/ Median (IQR)/N (%)***  ***N=1167*** |  |
| Age (years) | 31.33 (5.16) | 30.20 (5.58) | <0.001 |
| Multiparous | 162 (45.8) | 697 (59.8) | <0.001 |
| BMI (kg/m^2^) | 36.45 (5.16) | 36.25 (4.65) | 0.508 |
| Ethnicity |  |  |  |
| White | 251 (70.9) | 706 (60.5) | <0.001 |
| Black | 63 (17.8) | 325 (27.9) | <0.001 |
| Asian | 11 (3.1) | 80 (6.9) | 0.012 |
| Other | 29 (8.2) | 55 (4.7) | 0.013 |
| Current smoker in early pregnancy | 8 (2.3) | 97 (8.3) | <0.001 |
| Socioeconomic deprivation | 217 (78.3) | 790 (81.0) | 0.338 |
| Gestational diabetes* | 103 (29.1) | 274 (28.4) | 0.794 |
| Gestational weight gain (kg)** | 7.54 (4.55) | 7.48 (4.54) | 0.838 |
| Gestation at delivery (weeks) | 39.71 (38.71 to 40.86) | 40.00 (38.86 to 40.86) | 0.032 |
| Birthweight (grams) | 3500 (3165 to 3806) | 3450 (3100 to 3790) | 0.126 |
| Neonatal sum of skinfold thicknesses (mm)^ | 10.77 (2.78) | 11.02 (2.55) | 0.320 |
| Neonatal abdominal circumference (cm) | 32.22 (2.18) | 32.68 (1.98) | 0.016 |
| Neonatal arm circumference (cm) | 11.51 (0.99) | 11.46 (1.15) | 0.651 |
| **Gestational diabetes diagnosed using the International Association of Diabetes in Pregnancy Group’s criteria at 24-28 weeks’ gestation. **Gestational weight gain defined as total weight gain from calculated pre-pregnancy weight gain to 34-36 weeks’ gestation. ^Neonatal sum of skinfolds defined as sum of triceps skinfold thicknesses and subscapular skinfold thicknesses, each measured in triplicates.* | | | |
