# Supplementary material for: Mode of infant feeding, eating behaviour and anthropometry in infants at 6-months of age born to obese women – a secondary analysis of the UPBEAT trial
Source: BMC Pregnancy Childbirth. 2018 Sep 3;18:355. doi: 10.1186/s12884-018-1995-7 (PMC6122563; doi:10.1186/s12884-018-1995-7)
Supplement: Supplementary file 9 — Table S7. Sensitivity analysis of removal of infants born > 34 weeks’ gestation and ≤ 37 weeks’ gestation (n = 8 excluded). (DOCX 14 kb) [file 12884_2018_1995_MOESM9_ESM.docx]

| **Table S7: Sensitivity analysis of removal of infants born >34 weeks’ gestation and ≤37 weeks’ gestation (n=8 excluded).** | | | |
| --- | --- | --- | --- |
|  | **Breastfeeding** | **Formula feeding** | **Mixed feeding** |
|  |  | *Mean difference/ Odds ratio*  *(95% CI)* | *Mean difference/ Odds ratio*  *(95% CI)* |
| Triceps skinfold z-scores* | REF | -0.02 (-0.39 to 0.35) | 0.47 (-0.19 to 1.14) |
| Subscapular skinfold z-scores* | REF | 0.21 (-0.15 to 0.57) | 0.37 (-0.27 to 1.01) |
| Sum of skinfold thickness (mm)** | REF | 0.27 (-0.71 to 1.26) | 1.44 (-0.32 to 3.20) |
| Weight z-scores* | REF | 0.27 (0.01 to 0.52) | 0.23 (-0.23 to 0.69) |
| BMI z-scores* | REF | 0.21 (-0.24 to 0.66) | 0.54 (-0.27 to 1.34) |
| Length z-scores* | REF | 0.29 (-0.17 to 0.74) | -0.28 (-1.10 to 0.54) |
| Total body fat estimation (%) ^ | REF | 0.37 (-0.88 to 1.61) | 1.83 (-0.39 to 4.05) |
| Arm circumference z-scores * | REF | 0.09 (-0.18 to 0.35) | 0.54 (0.6 to 1.01) |
| Weight change (kg/ month) | REF | 0.04 (0.01 to 0.07) | 0.03 (-0.03 to 0.09) |
| Length change (cm/month) | REF | 0.16 (-0.06 to 0.38) | 0.01 (-0.36 to 0.38) |
| Catch up growth | REF | 2.40 (1.40 to 4.12) | 1.24 (0.46 to 3.31) |
| Catch down growth | REF | 0.67 (0.38 to 1.19) | 0.45 (0.13 to 1.50) |
| **Infant z-scores calculated using the WHO growth standards [24]. **Infant sum of skinfold thicknesses calculated as the addition of subscapular and triceps skinfolds thicknesses, each measured in triplicates. ^Infant total body fat estimation calculated sex-specific, validated equations [25]. †Analyses adjusted for randomisation to the UPBEAT Intervention, infant sex and infant age at anthropometric measurement as well as maternal early pregnancy BMI, ethnicity, socioeconomic deprivation, gestational diabetes and infant size at birth.* | | | |
