# Supplementary material for: Mode of infant feeding, eating behaviour and anthropometry in infants at 6-months of age born to obese women – a secondary analysis of the UPBEAT trial
Source: BMC Pregnancy Childbirth. 2018 Sep 3;18:355. doi: 10.1186/s12884-018-1995-7 (PMC6122563; doi:10.1186/s12884-018-1995-7)
Supplement: Supplementary file 10 — Table S8. Predictors of missing exposure (mode of infant feeding) and maternal covariate data in infants with detailed anthropometric data at 6 months of age. (DOCX 17 kb) [file 12884_2018_1995_MOESM10_ESM.docx]

| **Table S8: Predictors of missing exposure (mode of infant feeding) and maternal covariate data in infants with detailed anthropometric data at 6 months of age.** | | | | |
| --- | --- | --- | --- | --- |
|  | | **Missing data** | **Complete data** | **Comparison** |
|  |  | **N (%), Mean (SD)** | **N (%), Mean (SD)** |  |
|  |  | **N=345** | **N=353** |  |
| Centre | St Thomas’ | 100 (28.9) | 107 (30.4) | 0.021 |
|  | King’s College Hospital | 54 (15.9) | 53 (14.8) |  |
|  | Newcastle | 49 (14.2) | 71 (20.2) |  |
|  | Glasgow | 107 (30.9) | 77 (21.9) |  |
|  | Manchester | 13 (3.8) | 28 (8.0) |  |
|  | Bradford | 5 (1.4) | 7 (2.0) |  |
|  | Sunderland | 8 (2.3) | 5 (1.4) |  |
|  | St Georges’ | 9 (2.6) | 5 (1.4) |  |
| **Maternal demographic characteristics** | | | | |
| Age (years) | | 30.1 (5.70) | 31.6 (5.05) | 0.001 |
| Multiparity | | 181 (52.3) | 162 (46.0) | 0.097 |
| Ethnicity | White | 249 (72.0) | 249 (70.7) | 0.72 |
|  | Black | 71 (20.5) | 63 (17.9) |  |
|  | Asian | 14 (4.0) | 11 (3.1) |  |
|  | Other | 12 (3.5) | 29 (8.2) |  |
| Socioeconomic deprivation | | 200 (84.4) | 217 (78.9) | 0.11 |
| Number of years in full time education | | 14.5 (2.9) | 15.7 (2.7) | <0.001 |
| **Maternal anthropometry** | | | | |
| Early pregnancy BMI (kg/m^2^) | | 36.1 (4.5) | 36.4 (5.1) | 0.34 |
| Sum of skinfold thickness (mm) | 15-18 weeks’ gestation | 123.8 (27.0) | 122.7 (26.6) | 0.58 |
|  | 27-28^+6^ weeks’ gestation | 126.6 (25.1) | 124.0 (27.0) | 0.19 |
|  | 34-36 weeks’ gestation | 123.4 (26.7) | 122.8 (27.0) | 0.79 |
| Total gestational weight gain (kg)** | | 7.2 (4.6) | 7.6 (4.5) | 0.24 |
| **Antenatal clinical history** | | | | |
| Diagnosis of GDM* | | 96 (28.9) | 103 (29.3) | 0.92 |
| Pre-eclampsia | | 12 (3.5) | 10 (2.9) | 0.63 |
| **Birth outcomes** | | | | |
| Mode of delivery | Vaginal | 165 (47.7) | 170 (48.3) | 0.32 |
|  | Instrumental | 51 (14.7) | 369 (11.1) |  |
|  | C-section | 130 (37.6) | 143 (40.6) |  |
| Postpartum haemorrhage (>1l) | | 47 (13.7) | 59 (16.9) | 0.24 |
| Neonatal sex | Male | 173 (50.0) | 182 (51.7) | 0.65 |
|  | Female | 173 (50.0) | 170 (48.3) |  |
| Gestation at delivery (weeks) | | 39.4 (2.3) | 39.9 (1.5) | 0.004 |
| Birthweight (kg) | | 3.42 (0.61) | 3.59 (0.52) | 0.09 |
| Neonatal sum of skinfold thicknesses (mm)^ | | 10.8 (2.8) | 11.0 (2.5) | 0.44 |
| Admission to NICU | | 33 (9.5) | 22 (6.3) | 0.11 |
| *Gestational diabetes diagnosed using the International Association of Diabetes in Pregnancy Group’s criteria at 24-28 weeks’ gestation. **Gestational weight gain defined as total weight gain from calculated pre-pregnancy weight gain to 34-36 weeks’ gestation. ^Neonatal sum of skinfolds defined as sum of triceps skinfold thicknesses and subscapular skinfold thicknesses, each measured in triplicates | | | | |
