# Supplementary material for: Mode of infant feeding, eating behaviour and anthropometry in infants at 6-months of age born to obese women – a secondary analysis of the UPBEAT trial
Source: BMC Pregnancy Childbirth. 2018 Sep 3;18:355. doi: 10.1186/s12884-018-1995-7 (PMC6122563; doi:10.1186/s12884-018-1995-7)
Supplement: Supplementary file 11 — Table S9. Sensitivity analyses assessing the role of mode of early feeding on measures of infant anthropometry at 6 months of age., in offspring born to obese women (n = 353) using multiple imputation. (DOCX 15 kb) [file 12884_2018_1995_MOESM11_ESM.docx]

| **Table S9: Sensitivity analyses assessing the role of mode of early feeding on measures of infant anthropometry at 6 months of age., in offspring born to obese women (n=353) using multiple imputation.** | | | |
| --- | --- | --- | --- |
|  | **Breastfeeding (46.4%)** | **Formula feeding (46.0%)** | **Mixed feeding (7.6%)** |
|  |  | **Mean difference/ Odds ratio**  **(95% CI) **** | **Mean difference/ Odds ratio**  **(95% CI) **** |
| Triceps SFT z-scores* | REF | 0.13 (0.18 to 0.43) | 0.31 (-0.23 to 0.86) |
| Subscapular SFT z-scores* | REF | 0.03 (-0.23 to 0.34) | 0.12 (-0.43 to 0.66) |
| SSFT (mm) | REF | -0.27 (-1.11 to 0.57) | 0.42 (-1.05 to 1.89) |
| Total body fat estimation (%) | REF | -0.34 (-1.40 to 0.72) | 0.54 (-1.33 to 2.40) |
| Weight z-scores* | REF | 0.25 (0.06 to 0.44) ^ | 0.11 (-0.25 to 0.46) |
| BMI z-scores* | REF | -0.00 (-0.34 to 0.33) | 0.07 (-0.55 to 0.69) |
| Length z-scores* | REF | -0.37 (-0.49 to 0.75) | -0.04 (-1.36 to 0.38) |
| Arm circumference z-score | REF | 0.21 (-0.11 to 0.52) | 0.74 (-0.11 to 1.05) |
| Rate of weight gain (kg/month) | REF | 0.03 (0.01 to 0.06) ^ | 0.01 (-0.04 to 0.06) |
| Rate of length gain (cm/month) | REF | 0.31 (-0.14 to 0.75) | 0.27 (-0.07 to 0.61) |
| BMI z-scores ≥85^th^ * | REF | 1.06 (0.54 to 2.08) | 1.89 (0.63 to 5.68) |
| BMI z-scores ≥ 95^th^ * | REF | 1.10 (0.40 to 3.07) | 1.62 (0.32 to 8.26) |
| Catch up growth | REF | 2.06 (1.31 to 3.26) ^ | 1.15 (0.49 to 2.70) |
| Catch down growth | REF | 0.78 (0.49 to 1.26) | 0.66 (0.26 to 1.67) |
| *Infant z-scores calculated using the WHO growth standards [24]; Catch up and catch down growth defined using the WHO definitions of change in weight >0.67 SDs; Infant sum of skinfold thicknesses calculated as the addition of subscapular and triceps skinfolds thicknesses, each measured in triplicates. ^Infant total body fat estimation calculated using sex-specific, validated equations [25]. Multiple imputation methodology; data was imputed to create 50 datasets using 10 burn-in iterations for missing mode of feeding (exposure) and covariate data for infants with detailed anthropometry at 6 months of age (n=698, 49.2% missing) using centre, maternal age, trial entry BMI, parity, smoking status, randomisation allocation, ethnicity, socioeconomic deprivation, diagnosis of gestational diabetes, total gestational weight gain, sum of skinfold thicknesses at 15-18+6, 27-28+6 and 34-36 weeks’ gestation, mode of delivery, gestation at delivery, admission to neonatal intensive care, neonatal sex and offspring age at 6 month visit. Multivariate regression analyses (adjusted for potential confounders) was performed and effect estimates were estimate on pooled datasets using mean difference (continuous outcomes) and odds ratio (binary outcomes). **Adjustment made randomisation for the UPBEAT Intervention, infant sex and infant age at anthropometric measurement as well as maternal early pregnancy BMI, ethnicity, socioeconomic deprivation, gestational diabetes and infant size at birth. ^ p<0.05 | | | |
